# Evaluation of culturally tailored breast cancer education video in a primarily Hispanic population

**DOI:** 10.1016/j.pecinn.2026.100470

**Published:** 2026-03-13

**Authors:** Priyanka Dadha, Devon Gonzalez, Pracheta Matharasi, Navkiran Shokar, Jennifer Molokwu

**Affiliations:** a Molecular and Translational Medicine, Texas Tech University Health Sciences Center, El Paso, Paul L. Foster School of Medicine, El Paso, TX, United States of America; b Department of Family and Community Medicine, Texas Tech University Health Sciences Center, El Paso, Paul L. Foster School of Medicine, El Paso, TX, United States of America; c Department of Population Health, The University of Texas at Austin, Dell Medical School, United States of America

**Keywords:** Breast cancer screening, Hispanic women, Culturally tailored intervention, Mobile health (mHealth), Health belief model, Health education video

## Abstract

**Objective:**

To evaluate the effectiveness of an online, culturally tailored educational video in improving breast cancer knowledge, psychosocial constructs, and screening intentions among a predominantly Hispanic population.

**Methods:**

A pragmatic pre–post trial was conducted between August 2020 and July 2021 across the U.S., with focused outreach in a Hispanic-majority border city. Participants aged 21–78 years were recruited through community health workers, social media, and partner organizations. The 12-min bilingual video, guided by the Health Belief Model and Social Cognitive Theory, addressed breast cancer epidemiology, screening guidelines, and cultural misconceptions. Surveys administered before and after the intervention measured knowledge, perceived susceptibility, perceived benefits, perceived barriers, self-efficacy, and screening intentions. Paired *t*-tests or Wilcoxon signed-rank tests assessed pre–post differences (*p* ≤ 0.05).

**Results:**

Of 200 individuals who accessed the survey, 167 (83.5%) completed both assessments. Participants' mean age was 45.4 years (SD = 9.8); most were female (95.8%) and Hispanic/Latino (69.5%). Post-intervention analyses showed significant improvements in breast cancer knowledge (+7%; *p* < 0.002), perceived benefits (+4%; *p* < 0.02), self-efficacy (+4.5%; *p* < 0.0004), and screening intention (+8.1%; *p* < 0.01).

**Innovation:**

This study demonstrates the utility of a brief, theory-informed, bilingual mHealth video to address breast cancer screening disparities in Hispanic/Latina women. The culturally tailored digital format supports scalability and accessibility in underserved communities.

**Conclusions:**

A short, culturally adapted video improved breast cancer knowledge, self-efficacy, and screening intentions, suggesting potential for broader public health integration.

## Highlights

• mHealth video improved breast cancer knowledge in Hispanic/Latina women.
• Education increased self-efficacy and mammography screening intention.
• Health Belief Model outcomes support culturally tailored interventions.
• Digital, bilingual tools may reduce screening disparities in underserved groups.

## 1 Background

Breast cancer is one of the most commonly diagnosed cancers and the second leading cause of cancer-related death among women in the United States, following skin cancer. [1] Despite advances in treatment, it causes over 40,000 deaths annually in the US [2], [3]. Early detection through mammography has been shown to reduce breast cancer mortality significantly. [4] The incidence of invasive breast cancer continues to rise each year, attributed in part to hormonal influences, excess body weight, decreased fertility rates, and delayed age at first birth, factors that are increasingly prevalent among American women. [5] These reproductive trends may result in prolonged unopposed estrogen exposure, thereby stimulating cell proliferation and elevating cancer risk. [6] Simultaneously, mortality rates from breast cancer have declined since 1989 owing to advances in early detection and improved treatment strategies. [7]

Despite overall progress in breast cancer awareness and care, significant disparities in outcomes remain across racial and ethnic groups. Hispanic women have a 30% lower reported incidence of breast cancer compared to non-Hispanic White women [8]. However, while breast cancer mortality has declined by 39% among non-Hispanic White women, the reduction among Hispanic women is only 29%. [8] Although the overall incidence is lower in Hispanic women, lower screening rates contribute to later-stage diagnoses and worse outcomes. [9]

Numerous social, structural, and cultural barriers hinder breast cancer screening uptake among Hispanic women in the US, including higher poverty rates, limited access to healthcare, low health literacy, and psychosocial factors such as fear of diagnosis. [9] In 2019, 19.4% of Hispanic women lived in poverty, more than double the rate among non-Hispanic White women. [10] Additional barriers, such as inadequate transportation, a lack of childcare, inflexible work schedules, and the absence of health insurance, further exacerbate disparities. [11], [12]

Cultural influences also play a significant role. Language barriers and differences in communication styles between patients and providers can impede understanding and trust. [13] For instance, Hispanic patients may prefer directive medical advice, which can affect decision-making when such guidance is not communicated. [14] Family medical histories are often incomplete due to limited access or awareness among relatives, and disease-related stigma may discourage open communication within families. [13], [15] Furthermore, fatalistic beliefs grounded in religious or cultural perspectives may lead to the perception that cancer is predetermined and beyond human control, thereby reducing motivation for preventive screening. [13]

Several studies have evaluated mobile health (mHealth) and video-based interventions as promising tools for addressing these multifaceted barriers and improving cancer screening awareness and behaviors among underserved populations. [16], [17] The World Health Organization defines mHealth as “medical and public health practice supported by mobile devices.” Researchers have emphasized that the content and the mode of delivery influence the effectiveness of health communication. [18] When tailored to the unique social, cultural, and linguistic needs of the priority population, these digital tools can increase engagement and uptake of preventive services. [16] The Community Preventive Services Task Force has endorsed the use of small media, including videos and print materials, as effective tools for increasing cancer screening rates, particularly when tailored for specific populations. Such interventions overcome barriers, such as language, transportation, and scheduling, by delivering education in accessible formats. [19] Studies have shown that video-based approaches can be particularly effective in enhancing knowledge and adherence to screening recommendations. [20]

Therefore, this study aimed to evaluate the effectiveness of a culturally tailored online educational video in improving breast cancer knowledge, perceived susceptibility, self-efficacy, and screening intentions while reducing perceived barriers among a predominantly Hispanic population.

## 2 Methods

### 2.1 Study design and setting

This study used a pragmatic pre–post-trial design to evaluate the effectiveness of a culturally tailored breast cancer education video on psychosocial factors influencing breast cancer screening and participants' intention to undergo screening. The study was conducted across the United States, focusing on community outreach through digital platforms and localized recruitment efforts. A significant portion of the intervention was disseminated across XXX, including XXX, a predominantly Hispanic border city with a population of approximately 865,000, over 80% of whom identify as Hispanic or Latino. XXX faces several health-related social challenges, including high uninsured rates, lower levels of educational attainment, and elevated poverty levels. These factors contribute to disparities in access to preventive services such as breast cancer screening. The culturally tailored video and accompanying surveys were distributed online and through in-person efforts by community health workers (CHWs) to engage a diverse sample of these underserved communities. The study duration was from August 2020 to July 2021. Copies of the pre-and post-intervention surveys are provided in Supplementary Material S01.

### 2.2 Study sample

The study sample for this analysis included participants aged 21–78 across the United States. Participants accessed the survey on an accrual basis through community site flyers or through links shared on social media platforms. Inclusion criteria were: (1) adults aged 21 years or older, (2) ability to understand English or Spanish, and (3) willingness to provide informed consent and complete both the pre- and post-intervention surveys. Exclusion criteria included individuals younger than 21 years, those unable to provide informed consent, and participants who did not complete either the pre- or post-intervention survey.

Although routine breast cancer screening guidelines in the United States recommend mammography beginning at age 40 for average-risk individuals, participants aged 21–39 were intentionally included in this study. The intervention focused on breast cancer education rather than screening uptake alone, and inclusion of younger adults allowed assessment of early awareness, perceived susceptibility, and future screening intention. Early educational exposure may influence long-term preventive health behaviors and preparedness for timely screening later in life, particularly in underserved populations.

### 2.3 Intervention

The culturally tailored, bilingual educational video, which covers breast cancer epidemiology, risk factors, symptoms, and screening recommendations, was developed using the Health Belief Model (HBM) and Social Cognitive Theory. The development, implementation, and evaluation of this Breast Cancer Screening and Navigation (BEST) intervention have been previously reported by Molokwu et al. [21] In that study, the intervention was delivered in person by trained community health workers and demonstrated effectiveness in improving breast cancer knowledge, psychosocial determinants of screening, and navigation-related outcomes among Hispanic women. The current manuscript extends this prior work by evaluating the effectiveness of the educational component of the BEST intervention when delivered through digital and hybrid dissemination approaches. This extension was undertaken to assess the reach and feasibility of delivering breast cancer education beyond in-person navigation settings, particularly among underserved Hispanic communities facing barriers such as limited access to care, low health literacy, and lack of insurance.The educational material was developed with community input through focus groups, and the video aimed to enhance knowledge of screening, diagnosis, and treatment services, regardless of insurance status, while addressing barriers such as low health literacy, limited awareness of screening, and cultural misconceptions identified during the formative and implementation phases of the BEST intervention. [21] The intervention consisted of providing the educational video titled “Breast Cancer Education Screening Program,” developed to promote breast cancer awareness and screening among a predominately Hispanic population. The video content was designed to align with constructs of the HBM, emphasizing perceived susceptibility, perceived benefits, self-efficacy, and reducing perceived barriers. Topics included basic information on breast cancer, the significance of early detection, the recommended age and frequency for mammography, and a detailed explanation of what to expect during a screening appointment. It also addressed common cultural myths, fears, and misconceptions surrounding breast cancer and screening. The total runtime of the video was approximately 12 min, and it was shown to participants before administering the post-intervention survey.

### 2.4 Sampling protocol and measures

The video was shared through a link distributed on social media and collaborative organizational websites. At the same time, CHWs also conducted in-person recruitment by handing out flyers with QR codes. Embedded within the video delivery system were survey links that allowed participants to complete a pre-survey, view the video, and then complete the post-surveys on their devices. Participants received a $25 gift card upon completing the post-intervention survey.

The survey collected demographic variables, including age, race/ethnicity, educational attainment, country of birth, marital status (married or living with a partner), employment status, household income, and self-reported health status. Psychosocial constructs were evaluated using validated survey instruments including knowledge (13 items in total, with accurate, false, or don't know responses), [22] perceived susceptibility (3 item Likert scale scored from 1 to 5, Cronbachs α = 0.87), [23], [24] perceived benefits (6 items scale scored from 1 to 5, Cronbach's alpha = 0.75), [23], [24] perceived barriers (15 item Likert scale scored 1–5, Cronbach's α = 0.88), [23], [24] self-efficacy (5-item Likert-type scale ranging from Strongly Agree [5] to Strongly Disagree [1] Cronbachs α = 0.87). [23], [24] Intent to undergo breast cancer screening was measured using a five-point scale, ranging from “I am not thinking of getting a mammogram at all” to “I am sure I will get a mammogram.”

The survey underwent standard back-translation, where Spanish-validated versions were unavailable [24], to ensure linguistic and conceptual equivalency between the English and Spanish versions. [25] Data was obtained through Qualtrics and was exported to a Microsoft Excel file.

### 2.5 Statistical analysis

A total of 200 participants accessed the survey link, and 167 (83.5%) completed both the pre- and post-surveys. We performed summary statistics to describe participant demographics. Continuous variables were summarized using means and standard deviation (SD), while categorical variables were presented as frequencies and percentages. A two-sided *p*-value of ≤0.05 was considered statistically significant.

For the psychosocial constructs, composite scores were generated by calculating the mean of individual item responses. Correct responses were scored 1 for knowledge items, while incorrect or ‘don't know’ responses were scored 0. To evaluate the effectiveness of the intervention, paired *t*-test or Wilcoxon Sign rank test was used to compare pre- and post-intervention scores for each psychosocial variable. The primary outcomes included changes in knowledge, perceived susceptibility, perceived benefits, perceived barriers, self-efficacy, and intention to screen for breast cancer. All analyses were performed using IBM SPSS Statistics Version 25.0.

## 3 Results

A total of 167 participants who completed both surveys were included in the final analyses. The mean age of the participants was 45.4 years (SD = 9.8), ranging from 21 to 78 years. Most participants preferred English over Spanish (90.4% vs. 9.6%), identified as female (95.8%), and reported being married or living with a partner (79.0%). Nearly half were employed full-time (49.1%), with 32.3% reporting part-time employment.

Regarding ethnicity and race, 69.5% identified as Hispanic/Latino, and 91% identified as White, with over 60% reporting a household income of less than $50,000. 43.7% of the surveyed population had some college or vocational training.

Most participants (86.2%) self-reported their health status as good to excellent. Most participants were born in the United States (72.5%; 95% CI: 64.9–78.9); on average, they had lived there for 36.1 years (SD = 15.0). (Table 1).

**Table 1:** Demographic summary of the participants (*n* = 167) subjects included in the study.

Variables	n (%) /Mean/SD*	(95%CI)/Range*
Age (yrs) [mean (SD)(Range)]	45.4 (9.8) *	(21–78) *

Language Preference		
English	151 (90.4)	(84.7–94.2)
Spanish	16 (9.6)	(5.8–15.3)

Sex		
Female	160 (95.8)	(91.2–98.2)
Male	7 (4.2)	(1.8–8.9)
Married/Living with Partner	132 (79)	(71.9–84.8)

Employment Status		
Not Employed	31 (18.6)	(13.1–25.5)
Part-Time	54 (32.3)	(25.4–40.0)
Full-Time	82 (49.1)	(41.3–56.9)

Ethnicity		
Hispanic/Latino	116 (69.5)	(61.8–76.2)
Non-Hispanic/Latino	51 (30.5)	(23.8–38.2)

Race		
American Indian/Alaska Native	3 (1.8)	(0.5–5.6)
Asian	2 (1.2)	(0.2–4.7)
Black / African-American	10 (6)	(3.1–11.0)
White	152 (91)	(85.4–94.7)

Annual Household Income		
$0 - $50,000 per year	101 (60.5)	(52.6–67.9)
$50,001 - $100,000 per year	47 (28.1)	(21.6–35.7)
$100,000 + per year	13 (7.8)	(4.4–13.2)
Prefer not to answer	6 (3.6)	(1.5–8.0)

Education		
Any College/Voc. Degree	73 (43.7)	(36.1–51.6)
College Completed	38 (22.8)	(16.8–30.0)
Graduate Degree Completed	19 (11.4)	(7.2–17.4)
High School Completed	28 (16.8)	(11.6–23.5)
Less than High School	8 (4.8)	(2.2–9.5)
None	1 (0.6)	(0.03–3.8)

Self-Reported Health Status		
Poor/Fair	23 (13.8)	(9.1–20.2)
Good/Very Good/Excellent	144 (86.2)	(79.8–90.9)

Birth Country		
United States	121 (72.5)	(64.9–78.9)
Mexico	42 (25.7)	(18.9–32.6)
Other	3 (1.8)	(0.5–5.6)
Years Living in the US [mean (SD) (range)]	36.1 (15.0)*	(3–68)*

### 3.1 Psychosocial constructs and intervention outcomes

Statistically significant outcomes were observed in key psychosocial constructs following the viewing of the educational video. Participants demonstrated a significant increase in knowledge about breast cancer (7%; *p* < 0.002), perceived benefits of screening (4%; *p* < 0.02), and self-efficacy regarding breast cancer screening (4.5%; *p* < 0.0004). Additionally, participants showed a significant increase in their intention to complete a breast cancer screening via mammography post-intervention (8.1%; *p* < 0.01). No significant changes were observed in perceived barriers or perceived susceptibility to the disease. (Table 2).

**Table 2:** Impact of educational intervention on health beliefs and screening practices related to breast cancer (n = 167).

Construct	Pre-Test Score Mean (SD)	Post-Test Score Mean (SD)	Change in score Mean (SD) (%)	p-value
Knowledge	8.6 (1.65)	9.2 (1.79)	0.6 (6.64) (7.0)	<0.002⁎
Perceived barriers	33.6 (13.9)	32.9 (15.4)	−0.7 (−2.09) (−2.1)	<0.300
Perceived benefits	22.8 (4.8)	23.7 (4.4)	0.9 (3.96) (4.0)	<0.020⁎
Perceived susceptibility	7.7 (2.9)	7.9 (2.9)	0.2 (2.88) (2.6)	<0.200
Self-Efficacy	39.7 (8.0)	41.5 (7.7)	1.8 (4.47) (4.5)	<0.0004⁎
Intent to undergo screening	3.7 (1.2)	4.0 (1.2)	0.3 (9.16) (8.1)	<0.01⁎

⁎ *p* ≤ 0.05.

**Fig. 1:**
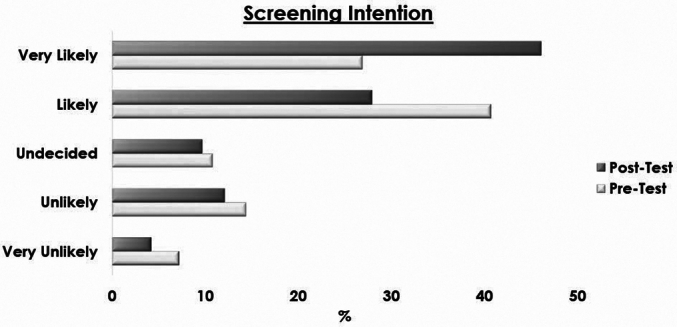
Changes in screening intentions before and after educational intervention.

Fig. 1 illustrates the notable improvement in intention to screen from pre- to post-intervention, confirming the positive impact of the culturally tailored video.

## 4 Discussion

This study found that viewing a culturally tailored breast cancer education video significantly improved participants' knowledge, perceived benefits, self-efficacy, and intention to obtain a mammogram for breast cancer screening. These findings are consistent with previous research indicating that culturally relevant interventions enhance understanding and motivation for preventive health behaviors. [16], [19] Particularly in an underserved Hispanic population. [26] The observed improvements align with the HBM, which posits that individuals are more likely to engage in health-promoting actions when they believe the benefits outweigh the barriers and have confidence in their ability to act. [27]

Knowledge significantly increased post-intervention, highlighting the effectiveness of culturally and linguistically appropriate educational content. Evidence supports that increasing knowledge of breast cancer screening improves outcomes among Hispanic women by empowering them to recognize early symptoms, understand the value of screening, and make informed health decisions. [16], [19], [28] Studies have demonstrated that health education interventions effectively increase breast cancer awareness and screening behaviors, particularly those tailored to specific cultural contexts. [16], [19] This study contributes to the growing body of evidence that knowledge delivery through digital tools, such as videos, can help bridge information gaps in medically underserved populations, particularly when community health literacy levels are low. [29], [30]

Perceived barriers to screening decreased slightly but not significantly, suggesting that while the intervention may have addressed informational and emotional barriers (e.g., fear or uncertainty), structural issues likely remained unaddressed. Previous literature has identified persistent barriers to breast cancer screening, including cost, access, language differences, low education and income, lack of insurance, being single, foreign-born, without a family history of cancer, and/or mistrust in healthcare providers. [31], [32], [33] A quasi-experimental study conducted at Menoufia University Hospital found that an educational intervention significantly improved breast self-examination practices, mammography adherence, interest in screening, and reduced perceived barriers. [34] Although our findings reflect a slight improvement, similar studies have shown that changes in perceived barriers require a more holistic approach, combining education with logistical support such as patient navigation, transportation assistance, or clinic-based interventions. [35] Therefore, future interventions should integrate these supportive elements to effectively reduce obstacles to screening adherence.

Perceived benefits showed a statistically significant increase post-intervention. This finding aligns with the HBM framework, which emphasizes the role of perceived benefits in motivating individuals to take action. When participants were informed about the advantages of early detection and the role of mammograms in reducing mortality, they were more likely to value screening. Prior studies have shown that emphasizing the positive outcomes of health behaviors enhances motivation [36] and promotes screening uptake, particularly among Hispanic women and other underserved populations with limited prior exposure to such health messages. [37], [38]

Perceived susceptibility to breast cancer showed a slight, non-significant increase following the intervention. This was not unexpected, as the educational video was designed to provide general breast cancer information rather than to influence personal risk perceptions. Risk perception is a multifaceted construct, shaped by cultural beliefs, family history, and prior experiences with the healthcare system, and may require more individualized or emotionally resonant approaches to meaningfully influence. While we did not assess the accuracy of participants' risk perceptions, this may be a more relevant outcome for future interventions. Prior studies have shown that Hispanic women, particularly older or Spanish-speaking individuals, often underestimate their breast cancer risk despite educational exposure. [39], [40], [41] Strategies such as storytelling, survivor testimonials, or personalized risk feedback may be necessary to meaningfully alter perceived susceptibility in this population.

Self-efficacy demonstrated a marked and significant improvement, suggesting that the video effectively enhanced participants' confidence in their ability to undergo screening. This is a critical outcome, as self-efficacy is one of the most robust predictors of preventive health behavior. When individuals believe they are capable of taking action, whether it's scheduling a mammogram or attending an appointment, they are more likely to follow through. [27], [42] This finding is particularly encouraging, as self-efficacy is a strong predictor of actual health behavior and a key determinant in breast cancer screening adherence. [27]

The positive result of this study may be attributed to the inclusion of step-by-step guidance in the video, which helps demystify the screening process and reduces anxiety about navigating the healthcare system. By improving participants' confidence in their ability to undergo screening, the intervention likely strengthened the foundation for long-term behavior change. Including actionable steps, such as how to schedule a mammogram or what to expect during the procedure, may have contributed to this positive outcome.

However, it is important to consider that behavioral intentions and self-efficacy may differ across age groups. For example, a 21-year-old participant may not be eligible for routine screening, and her confidence may primarily relate to knowledge acquisition or future planning. In contrast, women aged 50–78 are within the recommended screening age, and improvements in self-efficacy may translate more directly into immediate screening behavior. Future research could investigate these age-related differences by stratifying outcomes or tailoring interventions to specific age groups, allowing for a more nuanced understanding of how educational videos influence screening intentions and subsequent behavior across the lifespan.

The intention to undergo breast cancer screening significantly increased post-intervention, indicating readiness to engage in models such as the Theory of Planned Behavior. [43] This outcome is particularly encouraging, as increased intention is a strong predictor of subsequent screening uptake, especially when reinforced with reminders and support systems.

To support translation of intention into action, culturally and contextually tailored reminders and support systems may be necessary. These could include bilingual text message or phone call reminders about scheduling appointments, navigation assistance to identify nearby clinics offering low-cost or free mammography, and informational resources addressing common barriers such as transportation, childcare, or work schedule constraints. Community health workers (CHWs) could provide additional support by offering guidance on completing paperwork, understanding insurance coverage, or clarifying the screening process in culturally relevant ways. Prior research indicates that interventions incorporating such personalized follow-up and support are more effective at increasing actual screening rates among Hispanic women. [26], [44]

These findings suggest that while the video successfully improved knowledge, self-efficacy, and intention, pairing educational interventions with tailored reminders and support systems may further enhance screening uptake and help reduce disparities in breast cancer outcomes among Hispanic women.

This study contributes valuable evidence that mHealth interventions, particularly culturally and linguistically adapted videos, can be instrumental in addressing disparities. A study of 44,524 high-risk women found that both US and foreign-born Hispanic/Latina women were more likely to have outdated or no breast cancer screening than U.S.-born non-Hispanic White women. [45] Similarly, a NYC study (*n* = 666) showed low awareness of breast density, especially among Spanish speakers, foreign-born individuals, and those with less education. [46] These findings underscore the importance of targeted education and clear communication in enhancing screening-related knowledge, perceived risk, and self-efficacy among underserved populations.

To build on these findings, culturally tailored educational tools show promise; however, education alone may be insufficient to drive lasting behavioral change. Future interventions should integrate structural supports, such as cost reduction, transportation assistance, flexible scheduling, and community-based partnerships, to ensure a more holistic approach to cancer prevention. Expanding these efforts across diverse communities by utilizing multimedia materials embedded in community programs and clinical workflows, particularly in settings such as federally qualified health centers, can enhance accessibility and impact. Addressing practical barriers, such as lack of insurance, limited transportation, or inflexible work schedules, through patient navigation and financial assistance is essential. Longitudinal research is necessary to assess the sustained effects on screening behavior. Partnering with trusted community figures can further enhance cultural relevance, foster trust, and increase engagement in preventive care initiatives, such as breast cancer screening.

While this study benefited from a pragmatic design and the use of bilingual, culturally tailored materials that effectively engaged a diverse US population, several limitations should be acknowledged. The small, geographically limited sample and reliance on self-reported data introduce the potential for recall and social desirability biases. The short follow-up period limits conclusions about sustained behavior change, and the lack of follow-up data prevents confirmation of actual mammogram completion. Furthermore, the study did not assess participants' health literacy or prior exposure to breast cancer education, which may have influenced outcomes. The intervention may also not fully reflect the cultural diversity within Hispanic/Latino communities. Finally, selection bias is a possibility, as the analysis included only participants who completed both pre- and post-intervention surveys.

These limitations suggest several directions for future research. Longitudinal studies could investigate the extent to which improved behavioral intentions translate into actual screening behavior over time. Future interventions might integrate structural supports—such as cost reduction, transportation assistance, flexible scheduling, and patient navigation—to complement educational tools and facilitate sustained behavior change. Embedding multimedia educational materials within community programs and clinical workflows, including federally qualified health centers, could enhance accessibility and engagement. Partnering with trusted community figures may further increase cultural relevance and promote participation in preventive care initiatives, including breast cancer screening. Additionally, future studies could examine differences across age groups, health literacy levels, and prior exposure to cancer education to better tailor interventions to the needs of diverse Hispanic/Latino populations. Overall, addressing these limitations and exploring these avenues could strengthen the effectiveness, scalability, and cultural responsiveness of future breast cancer education interventions.

### 4.1 Innovation

This study contributes to the advancement of breast cancer screening interventions by isolating and evaluating the educational component of a culturally tailored navigation program rather than testing a bundled, multi-component intervention. Most prior interventions targeting Hispanic populations combine education with patient navigation, reminders, or structural supports, making it difficult to determine the independent contribution of culturally responsive education itself. By testing a stand-alone, theory-driven video derived from the BEST program, this study clarifies what psychosocial mechanisms can be activated through culturally tailored digital education alone. This approach refines assumptions about the role of education in disparity reduction and provides insight into how screening interventions can be structured in stepped or resource-limited settings.

The study also advances implementation strategies by translating a previously in-person, community health worker-delivered intervention into a scalable digital format and applying it within a predominantly Hispanic and U.S.-Mexico border context characterized by structural barriers to care. Rather than relying solely on clinical recruitment, the hybrid dissemination model, combining social media, QR-code outreach, and community engagement, demonstrates a pragmatic pathway for extending evidence-informed screening education beyond traditional healthcare settings. In addition, embedding validated psychosocial measures within the digital delivery platform provides a replicable framework for evaluating theory-based small-media interventions in underserved populations. Together, these contributions offer both conceptual and methodological innovation, advancing the design and scalable implementation of culturally responsive cancer prevention strategies.

## 5 Conclusions

This study demonstrated that a culturally tailored video intervention significantly enhanced key psychosocial factors related to breast cancer awareness: knowledge, perceived benefits of screening, self-efficacy, and intention to undergo mammography. These findings underscore the potential of culturally responsive and accessible educational tools to positively influence health behaviors, particularly in underserved communities facing structural and economic barriers. Continued investment in health literacy initiatives that promote preventive screening is essential to reducing disparities. The promising results support the use of culturally tailored videos as an effective strategy to improve screening intentions. Future research should incorporate strategies to address persistent perceived barriers and risk perceptions, include long-term follow-up to evaluate actual screening behavior, and expand outreach efforts to non-English speaking and lower-income populations to maximize public health impact.

## Abbreviations

**Table:** 

BC	Breast Cancer
US	United States
mHealth	Mobile Health
WHO	World Health Organization
CPSTF	Community Preventive Services Task Force
CHW	Community Health Worker
HBM	Health Belief Model
SD	Standard Deviation
SPSS	Statistical Package for the Social Sciences
CI	Confidence Interval

## Authors contribution

JM and NS conceived the study. PM developed the video intervention. DG conducted data analysis. PD contributed to manuscript writing and editing. All authors reviewed and approved the final manuscript.

## CRediT authorship contribution statement

**Priyanka Dadha:** Writing – review & editing, Conceptualization. **Devon Gonzalez:** Writing – original draft, Formal analysis. **Pracheta Matharasi:** Data curation. **Navkiran Shokar:** Investigation. **Jennifer Molokwu:** Writing – review & editing, Supervision, Conceptualization.

## Consent for publication

Participants provided consent for the publication of anonymized responses.

## Ethics approval and consent to participate

This study was approved by the Institutional Review Board at XXX (IRB# E17066) and Informed consent was obtained from all individual participants included in the study.

## Funding

This work was supported by a Cancer Prevention Program Grant from the 10.13039/100004917Cancer Prevention and Research Institute of Texas (CPRIT) (Grant number PP180003).

## Declaration of competing interest

The authors declare that they have no competing interests.

## Data Availability

The datasets generated during and/or analyzed during the current study are available from the corresponding author upon reasonable request.

## Appendix A Supplementary data

Supplementary material 1 Image 1

Supplementary material 2 Image 2

Supplementary material 3 Image 3

Supplementary material 4 Image 4
