# Supplementary material for: Evaluation of culturally tailored breast cancer education video in a primarily Hispanic population
Source: PEC Innov. 2026 Mar 13;8:100470. doi: 10.1016/j.pecinn.2026.100470 (PMC13022682; doi:10.1016/j.pecinn.2026.100470)
Supplement: Supplementary file 1 — Supplementary material 1 [file mmc1.docx]

# BEST Video Evaluation [Pre-Survey]

Name:

**last**

,

**first**

, ID:

**middle**

Date of Birth: / /

Telephone Number: Email Address:

Home Address

Street: City/County, State: Zipcode:


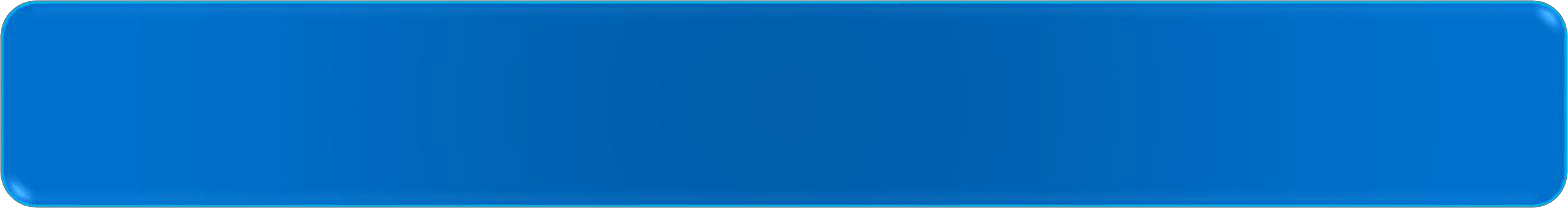

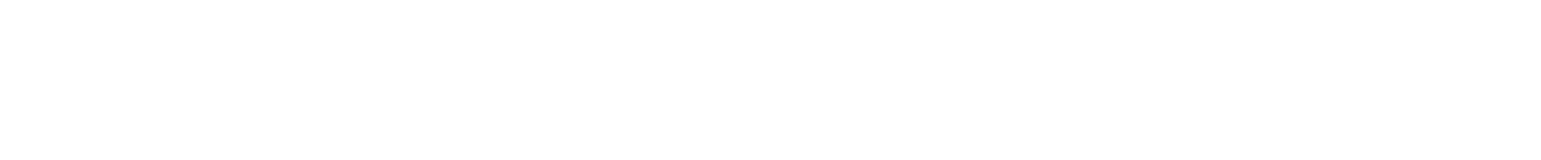


**The interview you are about to take should last about minutes. Although the questions that you will be answering today may seem repetitious at times, they will help us to better understand people’s thoughts and reactions to breast cancer and breast cancer screening . Please be assured that your responses will be kept confidential and you will not be personally identified in any reports or publication that may result from this study. You will be rewarded with a $25.00 gift card once completing both surveys and viewing the video in its entirety.**

**Please circle the numbers next to your answers. For example: ① Agree**

|  | **Section A. These statements are about breast cancer knowledge. Tell us whether you think they are true or false.** | | | | | | | |
| --- | --- | --- | --- | --- | --- | --- | --- | --- |
| Kn 1 | About 1 in 8 U.S. women will develop breast cancer over the course of her lifetime |  | True |  |  |  | False |  |
| kn2 | Breast cancer is the 3rd. most common cancer among American women |  | True | | |  | False | |
| Kn 3 | The average age to get breast cancer is around 60 years, but it does occur earlier and later as well |  | True |  |  |  | False | |
| Kn4 | If you get hit on your breast that you can get breast cancer | True | | | |  | False | |
| Kn5 | A woman who has her first child before the age of 35 is more likely to develop breast cancer than a woman who has her first child after the age of 35. | True | | | |  | False | |
| Kn6 | The constant irritation of a tight bra can, over time, cause breast cancer | True | | | |  | False | |
| Kn7 | A healthy diet ,breastfeed your children and regular exercise can reduce the risk for breast cancer | True | | | |  | False | |
| Kn8 | Mammography is recommended every 1 or 2 years between 50 and 75 years of age. | True | | | |  | False | |
| Kn9 | Minority women are less likely to be diagnosed at an earlier stage | True | | | |  | False | |
| Kn10 | The most common way breast cancer is found is when a woman notices a painless lump | True | | | |  | False | |
| Kn11 | Some people do not have any signs or symptoms of Breast Cancer until a later stage | True | | | |  | False | |
| Kn12 | In a mammogram results a dense breast means more fatty tissue in the breast –this means the X-rays could not easily see the breast | True | | | |  | False | |
| Kn13 | If a cyst is found in a mammogram you have more risk to develop breast cancer | True | | | |  | False | |

| **Section B The following are possible reasons why you might find it hard to get breast cancer screening. Please tell us how much each of these things affect your decision to get screening for breast cancer.** | | | | | | | | | | |
| --- | --- | --- | --- | --- | --- | --- | --- | --- | --- | --- |
|  | |  | |  | |  | **Strongly Agree** | | | |
|  |  |  | |  | **Agree** | |  |  |  |  |
|  |  |  | | **Undecided** | | | | |  |  |
|  |  |  |  | **Disagree** | |  |  |  |  |  |
|  |  | **Strongly Disagree** | | | | |  |  |  |  |
| **bar1.** | I am afraid to have a mammogram because I might find out something is wrong. |  | |  | | 1 | 2 | 3 | 4 | 5 |
| **bar2.** | I am afraid to have a mammogram because I don’t understand what will be done. |  | |  | | 1 | 2 | 3 | 4 | 5 |
| **bar3.** | Having a mammogram is too embarrassing. |  | |  | | 1 | 2 | 3 | 4 | 5 |
| **bar4.** | Having a mammogram exposes me to unnecessary radiation |  | |  | | 1 | 2 | 3 | 4 | 5 |
| **bar5.** | I don’t know how to get a mammogram if I don’t have medical insurance |  | |  | | 1 | 2 | 3 | 4 | 5 |
| **bar6.** | I don’t have time to get a mammogram |  | |  | | 1 | 2 | 3 | 4 | 5 |
| **bar7.** | I think I don’t need to get a mammogram |  | |  | | 1 | 2 | 3 | 4 | 5 |
| **bar8.** | I kept putting it off mammography screening |  | |  | | 1 | 2 | 3 | 4 | 5 |
| **bar9.** | I didn't know I needed one |  | |  | | 1 | 2 | 3 | 4 | 5 |
| **bar10.** | My doctor didn't tell me I needed one |  | |  | | 1 | 2 | 3 | 4 | 5 |
| **bar11.** | I don’t have transportation to get a mammogram |  | |  | | 1 | 2 | 3 | 4 | 5 |
| **bar12.** | I don’t have immigration documents |  | |  | | 1 | 2 | 3 | 4 | 5 |
| **bar13.** | I have difficulty to obtain an interpreter |  | |  | | 1 | 2 | 3 | 4 | 5 |
| **bar14.** | I’m afraid of discriminatory treatment because of my race or ethnic group |  | |  | | 1 | 2 | 3 | 4 | 5 |
| **bar15.** | I am afraid of the mammogram procedure |  | |  | | 1 | 2 | 3 | 4 | 5 |

| **Section C The following statements are about the benefits that you may experience from getting breast cancer screening. Please tell us if you agree or disagree with the following statements.** | | | | | | | | | | |
| --- | --- | --- | --- | --- | --- | --- | --- | --- | --- | --- |
|  | |  | |  | |  | **Strongly Agree** | | | |
|  |  |  | |  |  | **Agree** |  |  |  |  |
|  |  |  | | **Undecided** | | | | |  |  |
|  |  |  |  | **Disagree** | |  |  |  |  |  |
|  |  | **Strongly Disagree** | | | | |  |  |  |  |
| **ben1.** | If I get a mammogram and nothing is found, I will not worry as much about breast cancer |  | |  | | 1 | 2 | 3 | 4 | 5 |
| **ben2.** | Having a mammogram will help me find breast lumps early |  | |  | | 1 | 2 | 3 | 4 | 5 |
| **ben3.** | If I find a lump through a mammogram, my treatment for breast cancer may not be as bad. |  | |  | | 1 | 2 | 3 | 4 | 5 |
| **ben4.** | Having a mammogram is the best way for me to find a very small lump. |  | |  | | 1 | 2 | 3 | 4 | 5 |
| **ben5.** | Having a mammogram will decrease my chances of dying from breast cancer. |  | |  | | 1 | 2 | 3 | 4 | 5 |
| **fcben6.** | Breast cancer survival can be improved if one participates in screening and early detection. |  | |  | | 1 | 2 | 3 | 4 | 5 |

| **Section D. The following statements are about how likely you feel or are worried to get breast cancer. Please tell us how you feel about these statements.** | | | | | | | | | | |
| --- | --- | --- | --- | --- | --- | --- | --- | --- | --- | --- |
|  | |  | |  | |  | **Strongly Agree** | | | |
|  |  |  | |  | **Agree** | | |  |  |  |
|  |  |  | | **Undecided** | | | | |  |  |
|  |  |  |  | **Disagree** | |  |  |  |  |  |
|  |  | **Strongly Disagree** | | | | |  |  |  |  |
| **sus1** | It is likely that I will get breast cancer |  | |  | | 1 | 2 | 3 | 4 | 5 |
| **sus2** | My chances of getting breast cancer in the next few years are great |  | |  | | 1 | 2 | 3 | 4 | 5 |
| **sus3** | I feel I will get breast cancer sometime during my life. |  | |  | | 1 | 2 | 3 | 4 | 5 |

| **Section E. The next statements are about completing the necessary steps to getting breast cancer screening. Please tell us if you agree or disagree with the following statements.** | | | | | | | | | | |
| --- | --- | --- | --- | --- | --- | --- | --- | --- | --- | --- |
|  | |  | |  | |  | **Strongly Agree** | | | |
|  |  |  | |  |  | **Agree** |  |  |  |  |
|  |  |  | | **Undecided** | | | | |  |  |
|  |  |  |  | **Disagree** | |  |  |  |  |  |
|  |  | **Strongly Disagree** | | | | |  |  |  |  |
| se1. | You can arrange transportation to get a mammogram |  | |  | | 1 | 2 | 3 | 4 | 5 |
| se2. | You can arrange other things in your life to have a mammogram |  | |  | | 1 | 2 | 3 | 4 | 5 |
| se3. | You can talk to people at the mammogram center about your concerns |  | |  | | 1 | 2 | 3 | 4 | 5 |
| se4. | You can get a mammogram even if you are worried |  | |  | | 1 | 2 | 3 | 4 | 5 |
| se5. | You can get a mammogram even if you don’t know what to expect |  | |  | | 1 | 2 | 3 | 4 | 5 |
| se6. | You can find a way to pay for a mammogram. |  | |  | | 1 | 2 | 3 | 4 | 5 |
| Se7. | You can make an appointment for a mammogram |  | |  | |  |  |  |  |  |
| Se8. | You know for sure you can get a mammogram if you really want to |  | |  | |  |  |  |  |  |
| Se9. | You know how to get a mammogram |  | |  | |  |  |  |  |  |
| Se10. | You can find a place to have a mammogram |  | |  | |  |  |  |  |  |

| **Section D.** **The next statements are about your plans to be tested for breast cancer with a mammogram**  **. Circle one of the following.** | | |
| --- | --- | --- |
| **Int 1.** | My plans for getting a mammogram in the next 6 months are | 1. I am not thinking of getting a mammogram at all 2. I think I need to consider getting a mammogram 3. I think I should get a mammogram, but I am not quite ready. 4. I think I will probably get a mammogram. 5. I am sure I will get a mammogram. |

| **Demographics**  **Below are some general questions about you. Remember, all of your answers are confidential.** | | | | | | | | | |
| --- | --- | --- | --- | --- | --- | --- | --- | --- | --- |
| **dem1.** | Age | yrs. | | | | | | | |
| **dem2.** | Gender |  Male | |  Female | | | |  Other | |
| **dem3.** | Are you married or living with a partner? |  Yes | | | |  No | | | |
| **dem4.** | Are you currently working? |  No | |  Yes [part-time] | | | |  Yes [full-time] | |
| **dem5.** | What is your annual household income from all sources? |  $0 - $20,000 per year   $20,001 - $50,000 per year   $50,001 - $100,000 per year   $100,000 + per year   Prefer not to answer | | | | | | | |
| **dem6.** | What is the highest grade/level of education that you have completed in any country? |  None:   Less than High School:   High School Completed:   Any College/Vocational Degree:   College Completed:  Graduate Degree Completed: | | | | | | | |
| **dem7.** | In general, would you say that your health is? |  Excellent |  Very Good | |  Good | |  Fair | |  Poor |
| **dem8.** | In what country were you born? |  United States | |  Mexico | | | |  Other: | |
| **dem9.** | How many years have you lived in the United States: | yrs. | | | | | | | |
| **dem10.** | Which one of the following would you say is your ethnicity? |  Hispanic / Latino | | | |  Non-Hispanic / Latino | | | |
| **dem11.** | Which one of the following would you say is your race? |  White   Asian   Black / African-American   American Indian / Alaska Native   Native Hawaiian / Pacific Islander | | | | | | | |

**Thank you so much for completing this survey!**
