# Supplementary material for: Evaluation of culturally tailored breast cancer education video in a primarily Hispanic population
Source: PEC Innov. 2026 Mar 13;8:100470. doi: 10.1016/j.pecinn.2026.100470 (PMC13022682; doi:10.1016/j.pecinn.2026.100470)
Supplement: Supplementary file 3 — Supplementary material 3 [file mmc3.docx]

# BEST Video Evaluation [Pre-Survey] Spanish

Nombre:

**Apellido**

,

**Nombre**

, ID:

**2o nombre**

Fecha de Ncimiento: / /

NUmero de telefono: correo electronico:


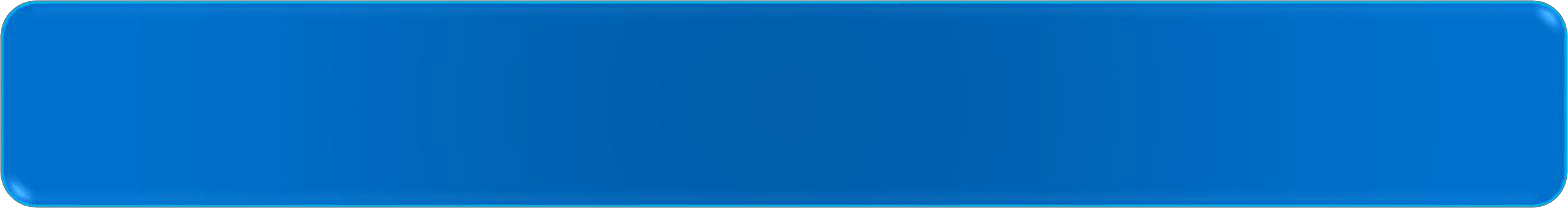

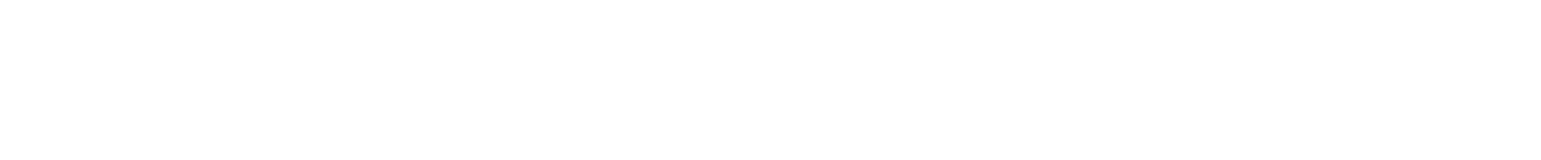


**La encuesta que está a punto de realizar debería durar unos_____ minutos. Aunque las preguntas que responderá hoy pueden parecer repetitivas, nos ayudarán a comprender mejor los pensamientos y reacciones de las personas al cáncer de mama y a las pruebas de detección del cáncer de mama. Tenga la seguridad de que sus respuestas se mantendrán confidenciales y no se lo identificará personalmente en ningún informe o publicación que pueda resultar de este estudio. Se le recompensará con una tarjeta de regalo de $ 25.00 una vez que complete ambas encuestas y vea el video en su totalidad.**

Home Address

Calle: Ciudad/Estado: codigo postal:

|  | **Sección A. Estas declaraciones se refieren al conocimiento del cáncer de mama. Díganos si cree que son verdaderos o falsos.** | | | |
| --- | --- | --- | --- | --- |
| Kn 1 | Aproximadamente 1 de cada 8 mujeres en Estados Unidos desarrollará cáncer de mama en el transcurso de su vida | 1. Verdadero |  | 1. Falso |
| kn2 | El cáncer de mama es el 3er cáncer más común entre las mujeres Americanas. | 1. Verdadero |  | 1. Falso |
| Kn 3 | La edad promedio para desarrollar el cáncer de mama es alrededor de los 60 años, pero puede ocurrir antes o después también. | 1. Verdadero |  | 1. Falso |
| Kn4 | Recibir un golpe en las mamas puede provocar cáncer de mama | 1. Verdadero |  | 1. Falso |
| Kn5 | Una mujer que tuvo su primer hijo antes de los 35 años tiene mas probabilidades de desarrollar cáncer de mama que una mujer que tuvo su hijo despues de los 35 años | 1. Verdadero |  | 1. Falso |
| Kn6 | La irritación constante por usar un brassiere ajustado puede causar después de un tiempo cáncer de mama. | 1. Verdadero |  | 1. Falso |
| Kn7 | Una dieta saludable, amamantar a tu hijo y hacer ejercicio regularmente puede reducir el riesgo de cáncer de mama | 1. Verdadero |  | 1. Falso |
| Kn8 | Las mujeres de 50 a 75 años deberán realizarse su exámen de mamografía cada 1 ó 2 años. | 1. Verdadero |  | 1. Falso |
| Kn9 | Las mujeres de las minorías tienen menos probabilidades de ser diagnosticadas en una etapa inicial. | 1. Verdadero |  | 1. Falso |
| Kn10 | La forma más común en que se detecta el cáncer de mama es cuando una mujer nota un bulto no doloroso | 1. Verdadero |  | 1. Falso |
| Kn11 | Algunas personas no presentan signos o síntomas de cáncer de mama hasta una etapa avanzada | 1. Verdadero |  | 1. Falso |
| Kn12 | En los resultados de una mamografía, una mama densa significa más tejido graso en la mama; esto significa que en las radiografías no puede ver fácilmente la mama | 1. Verdadero |  | 1. Falso |
| Kn13 | Si a alguien se le encuentra un quiste en una mamografía, tiene más riesgo de desarrollar cáncer de mama | 1. Verdadero |  | 1. Falso |

| **Sección B Las siguientes son posibles razones por las que podría resultarle difícil hacerse una prueba de detección del cáncer de mama. Díganos en qué medida cada una de estas cosas afecta su decisión de hacerse una prueba de detección del cáncer de mama.** | | | | | | | | | | |
| --- | --- | --- | --- | --- | --- | --- | --- | --- | --- | --- |
|  | |  | |  | |  | **Totalmente de acuerdo** | | | |
|  |  |  | |  | **De acuerdo** | | | | |  |
|  |  |  | | **Indeciso** | | | | |  |  |
|  |  |  | **En desacuerdo** | | | | |  |  |  |
|  |  | **Totalmente en desacuerdo** | | | | |  |  |  |  |
| **bar1.** | Me da miedo hacerme una mamografía porque puede que me encuentren algo malo. | | | | | 1 | 2 | 3 | 4 | 5 |
| **bar2.** | Tengo miedo de hacerme una mamografía porque no sé lo que me van a hacer. | | | | | 1 | 2 | 3 | 4 | 5 |
| **bar3.** | Me da mucha vergüenza que me hagan una mamografía. |  | |  | | 1 | 2 | 3 | 4 | 5 |
| **bar4.** | Hacerme una mamografía me expone a una radiación innecesaria. | | |  | | 1 | 2 | 3 | 4 | 5 |
| **bar5.** | No sé cómo hacerme una mamografía si no tengo seguro médico | | | | | 1 | 2 | 3 | 4 | 5 |
| **bar6.** | No tengo tiempo para hacerme una mamografía |  | |  | | 1 | 2 | 3 | 4 | 5 |
| **bar7.** | Creo que no necesito hacerme una mamografía |  | |  | | 1 | 2 | 3 | 4 | 5 |
| **bar8.** | He estado postponiendo hacerme una mamografia |  | |  | | 1 | 2 | 3 | 4 | 5 |
| **bar9.** | No sabía que necesitaba una |  | |  | | 1 | 2 | 3 | 4 | 5 |
| **bar10.** | Mi médico no me dijo que necesitaba una |  | |  | | 1 | 2 | 3 | 4 | 5 |
| **bar11.** | No tengo transporte para hacerme una mamografía |  | |  | | 1 | 2 | 3 | 4 | 5 |
| **bar12.** | No me la hago porque no tengo documentos de inmigración |  | |  | | 1 | 2 | 3 | 4 | 5 |
| **bar13.** | Tengo dificultad para conseguir un intérprete |  | |  | | 1 | 2 | 3 | 4 | 5 |
| **bar14.** | Tengo miedo de un trato discriminatorio debido a mi raza o grupo | étnico | |  | | 1 | 2 | 3 | 4 | 5 |
| **bar15.** | Tengo miedo del procedimiento de la mamografía |  | |  | | 1 | 2 | 3 | 4 | 5 |

| **Sección C Las siguientes declaraciones se refieren a los beneficios que puede experimentar al hacerse una prueba de detección del cáncer de mama. Díganos si está de acuerdo o en desacuerdo con las siguientes declaraciones.** | | | | | | | | | | |
| --- | --- | --- | --- | --- | --- | --- | --- | --- | --- | --- |
|  | |  | |  | |  | **Totalmente de acuerdo** | | | |
|  |  |  | |  | **De acuerdo** | | | |  |  |
|  |  |  | | **Indeciso** | | | | |  |  |
|  |  |  | **En desacuerdo** | | | | |  |  |  |
|  |  | **Totalmente en desacuerdo** | | | | |  |  |  |  |
| **ben1.** | Si me hacen una mamografía y no me encuentran nada, no me preocupará tanto el cáncer de mama. |  | |  | | 1 | 2 | 3 | 4 | 5 |
| **ben2.** | Hacerme una mamografía me ayudará a encontrar bultos o bolitas en el pecho con más facilidad. |  | |  | | 1 | 2 | 3 | 4 | 5 |
| **ben3.** | Si me encuentran un bulto o bolita en una mamografía, el tratamiento para el cáncer de mama puede que no sea tan malo. |  | |  | | 1 | 2 | 3 | 4 | 5 |
| **ben4.** | Hacerme una mamografía es la mejor manera de que me encuentren un bulto o bolita muy pequeño (a). |  | |  | | 1 | 2 | 3 | 4 | 5 |
| **ben5.** | Al hacerme una mamografía, tendré menos probabilidad de morir de cáncer de mama. |  | |  | | 1 | 2 | 3 | 4 | 5 |
| **fcben6.** | La supervivencia al cáncer de mama se puede mejorar si se realiza la prueba de detección o mamografia para un diagnostico temprano |  | |  | | 1 | 2 | 3 | 4 | 5 |

| **Sección D. Las siguientes declaraciones se refieren a que tanto esta Ud preocupada sobre su probabilidad de tener cáncer de mama. Díganos cómo se siente acerca de estas declaraciones** | | | | | | | | | | |
| --- | --- | --- | --- | --- | --- | --- | --- | --- | --- | --- |
|  | |  | |  | |  | **Totalmente de acuerdo** | | | |
|  |  |  | |  | **De acuerdo** | | | | |  |
|  |  |  | | **Indeciso** | | | | |  |  |
|  |  |  | **En desacuerdo** | | | | |  |  |  |
|  |  | **Totalmente en desacuerdo** | | | | |  |  |  |  |
| **sus1** | Es probable que llegue a tener cáncer de mama. |  | |  | | 1 | 2 | 3 | 4 | 5 |
| **sus2** | La probabilidad de que yo tenga un cáncer de mama en los próximos años es muy alta. |  | |  | | 1 | 2 | 3 | 4 | 5 |
| **sus3** | Creo que tendré cáncer de mama en algún momento de mi vida. |  | |  | | 1 | 2 | 3 | 4 | 5 |

| **Sección E. Las siguientes declaraciones se refieren a los pasos necesarios para hacerse la prueba de detección del cáncer de mama. Díganos si está de acuerdo o en desacuerdo con las siguientes declaraciones.** | | | | | | | | | | |
| --- | --- | --- | --- | --- | --- | --- | --- | --- | --- | --- |
|  | |  | |  | |  | **Totalmente de acuerdo** | | | |
|  |  |  | |  | **De acuerdo** | | | |  |  |
|  |  |  | | **Indeciso** | | | | |  |  |
|  |  |  | **En desacuerdo** | | | | |  |  |  |
|  |  | **Totalmente en desacuerdo** | | | | |  |  |  |  |
| se1. | Usted puede organizar su transporte para ir a realizarse una mamografía. |  | |  | | 1 | 2 | 3 | 4 | 5 |
| se2. | Usted puede organizar otras cosas en su vida para hacerse una mamografía. |  | |  | | 1 | 2 | 3 | 4 | 5 |
| se3. | Usted puede hablar con la gente en el centro de mamografía acerca de sus preocupaciones. |  | |  | | 1 | 2 | 3 | 4 | 5 |
| se4. | Puede hacerse una mamografía, incluso si usted está preocupada. |  | |  | | 1 | 2 | 3 | 4 | 5 |
| se5. | Puede hacerse una mamografía, incluso si usted no sabe qué esperar. |  | |  | | 1 | 2 | 3 | 4 | 5 |
| se6. | Usted puede encontrar la manera de pagar por una mamografía. |  | |  | | 1 | 2 | 3 | 4 | 5 |
| Se7. | Usted puede hacer una cita para una mamografía. |  | |  | | 1 | 2 | 3 | 4 | 5 |
| Se8. | Usted sabe con seguridad que puede hacerse una mamografía |  | |  | | 1 | 2 | 3 | 4 | 5 |
| Se9. | Si de verdad quiere, ya sabe que hacer para obtener una mamografía. |  | |  | | 1 | 2 | 3 | 4 | 5 |
| Se10. | Usted puede encontrar un lugar para hacerse una mamografía |  | |  | | 1 | 2 | 3 | 4 | 5 |

| **Sección F. Las siguientes declaraciones se refieren a sus planes para hacerse la prueba del cáncer de mama con una mamografía.**  **. Encierra en un círculo uno de los siguientes.** | | |
| --- | --- | --- |
| **Int 1.** | Mis planes para hacerme una mamografía en los próximos 6 meses son | 1. No estoy pensando en hacerme una mamografía. 2. Creo que debo considerar hacerme una mamografía 3. Creo que debería hacerme una mamografía, pero todavia no estoy lista. 4. Creo que probablemente me haré una mamografía. 5. Tengo la determinación de hacerme una mamografía. |

| **Demografia G**  **A continuación se presentan algunas preguntas generales sobre usted. Recuerde, todas sus respuestas son confidenciales.** | | | | | | | | | |
| --- | --- | --- | --- | --- | --- | --- | --- | --- | --- |
| **dem1.** | Edad | años. | | | | | | | |
| **dem2.** | Género |  Hombre | |  Mujer | | | |  Otro | |
| **dem3.** | ¿Está Ud. casado o vive con una pareja? |  Si | | | |  No | | | |
| **dem4.** | ¿ Esta actualmente trabajando? |  No | |  Si [medio-tiempo] | | | |  Si [tiempo completo] | |
| **dem5.** | ¿Cuál es su ingreso familiar anual de todas las fuentes? |  $0 - $20,000 por año   $20,001 - $50,000 por año   $50,001 - $100,000 por año   $100,000 + por año   Prefiero no contestar | | | | | | | |
| **dem6.** | ¿Cuál es el grado / nivel de educación más alto que ha completado en cualquier país? |  Ninguno   Menor de preparatoria o Preparatoria incompleta   Preparatoria Completa:   Universidad incomplete /Carrera técnica   Universidad Completa:  Post -grado completo | | | | | | | |
| **dem7.** | En general, ¿diría que su salud es? |  Excelente |  Muy Buena | |  Buena | |  regular | |  Mala |
| **dem8.** | ¿En que pais nacio? |  Estados Unidos | |  Mexico | | | |  Otro: | |
| **dem9.** | ¿Cuántos años ha vivido en los Estados Unidos? | años. | | | | | | | |
| **dem10.** | ¿Cuál de los siguientes diría que es su origen étnico? |  Hispanic / Latino | | | |  Non-Hispanic / Latino | | | |
| **dem11.** | ¿Cuál de las siguientes dirías que es tu raza? |  Blanco   Asiático   Negro / Afroamericano   Indio americano / nativo de Alaska   Nativo de Hawái / de las islas del Pacífico | | | | | | | |

**¡Muchas gracias por completar esta encuesta!**
